# The First Salamander Defensin Antimicrobial Peptide

**DOI:** 10.1371/journal.pone.0083044

**Published:** 2013-12-30

**Authors:** Ping Meng, Shilong Yang, Chuanbin Shen, Ke Jiang, Mingqiang Rong, Ren Lai

**Affiliations:** 1 Life Sciences College of Nanjing Agricultural University, Nanjing,Jiangsu, China; 2 Key Laboratory of Animal Models and Human Disease Mechanisms of Chinese Academy of Sciences and Yunnan Province, Kunming Institute of Zoology, Kunming, Yunnan, China; 3 State Key Laboratory of Genetic Resources and Evolution, Kunming Institute of Zoology, Chinese Academy of Sciences, Kunming, Yunnan, China; University of South Florida College of Medicine, United States of America

## Abstract

Antimicrobial peptides have been widely identified from amphibian skins except salamanders. A novel antimicrobial peptide (CFBD) was isolated and characterized from skin secretions of the salamander, *Cynops fudingensis*. The cDNA encoding CFBD precursor was cloned from the skin cDNA library of *C. fudingensis*. The precursor was composed of three domains: signal peptide of 17 residues, mature peptide of 41 residues and intervening propeptide of 3 residues. There are six cysteines in the sequence of mature CFBD peptide, which possibly form three disulfide-bridges. CFBD showed antimicrobial activities against *Staphylococcus aureus*, *Bacillus subtilis*, *Candida albicans* and *Escherichia coli*. This peptide could be classified into family of β-defensin based on its seqeuence similarity with β-defensins from other vertebrates. Evolution analysis indicated that CFBD was close to fish β-defensin. As far as we know, CFBD is the first β-defensin antimicrobial peptide from salamanders.

## Introduction

Antimicrobial peptides (AMP) play a critical role as defense molecules to protect animals from the invasion of bacteria, viruses or fungi [1], [2]. The cationic antimicrobial peptide defensins are one of the largest and most studied families of antimicrobial peptides. Up to now, more than 300 defensins have been identified. They represented in a range of organisms such as mammals, birds, invertebrates, plants and recently found in the ebonycup fungus [3], [4], but seldom found in amphibian especially in urodela. Defensins are usually consisted of 18–45 amino acids including six or eight conserved cysteine residues. They could be divided into three groups including: α-defensins, β-defensins and θ-defensins based on the different disulfide motif. The disulfide motif of β-defensins is C1–C5, C2 –C4 and C3–C6 [5], [6].

The moist skin of salamander usually makes them reliant on habitats in or near water or in a wetland [7]. There are some symbiotic bacteria on salamander skins, which possibly protect salamanders from microorganism infection. An epibiotic bacterium, *Lysobacter gummosus* (AB161361), was found on the skin of redback salamander, *Plethodon cinereus*. The beneficial bacteria produced a chemical antibiotic 2, 4-diace tylphloroglucinol, which could inhibit pathogenic fungi with IC_50_ value of 8.73 µM [8]. *Cutaneous bacterium* was another beneficial bacterium from skin of redback salamander. It reduced the community of *Batrachochytrium dendrobatidis* and protected the salamander from the lethal and sublethal effects of chytridiomycosis [9]. In addition, there is chemical array acting as defensive roles on salamander skins. Their skin glands secrete poison and antibiotics to protect themselves from predators and against infection of microbial pathogens [10]. F15, the only reported antimicrobial peptide from *P.cinereus*, showed strong activity against *S. aureus* [11]. It may be one of the reasons why this salamander was rarely found exhibiting infections.

In this study, we examined the skin of *C. fudingensi* for the existence of antimicrobial peptides. A novel β-defensin antimicrobial peptide was identified from the skin of salamander.

## Materials and Methods

### Ethics statement

The salamanders of *C. fudingensis* were collected in Fujian Province of China. Salamander, *C. fudingensis*, is not the protected species in China. The collection was under the permission of laotaishan natural protection park in funding (Frist we sent a application including the numbers and age of salamanders to administration section of laotaishan natural protection park.Then their sent back a permission to my institution. After that, we went to laotaishan and captured salamanders with staffs of the park). All procedures about salamanders were also approved by the Animal Care and Use Committee of Kunming Institute of Zoology. Experimental protocols using rabbit red cells in this work were approved by the Animal Care and Use Committee of Kunming Institute of Zoology. Approval to conduct these studies of human red cell was obtained from the ethics committee of the Institutional Review Board of the Kunming Institute of Zoology, Chinese Academy of Sciences. Human red cells were from volunteer shilong yang, who is also the author of this manuscript. He signed the informed consent for this study.

### Collection of salamander skin secretions

Adult salamanders of *C. fudingensis* were collected with a net in Fujian Province of China. Salamanders were put in a cylinder container. Then, skin secretions were collected manually by stimulating the skin of salamander using a 3 V alternating current for 3–5 s [12]. Skin secretions were washed with 0.1 M phosphate-buffered solution containing protease inhibitor mixture (sigma). The collected solutions containing skin secretions were quickly centrifuged (10000 rpm, 10 min). The supernatant were lyophilized and stored at −20°C for further using.

### Peptide purification

Lyophilized skin secretion sample of *C. fudingensis* was dissolved in phosphate buffer (0.1 M, pH 6.0, containing 5 mM EDTA, PBS). The sample was first separated by Sephadex G-50 (Superfine, GE Healthcare, 2.6 cm diameter and 100 cm length) gel filtration column equilibrated and eluted with 0.1 M phosphate buffer, pH 6.0. Elution was monitored at 280 nm and each fraction was 3.0 ml. Fractions containing antimicrobial activity were further purified using C_18_ reverse-phase high-performance liquid chromatography (RP-HPLC; Gemini C_18_ column, 5 µm particle size, 110 Å pore size, 250×4.6 mm). The buffers used for RP-HPLC were 0.1% (v/v) trifluoroacetic acid/water (Buffer A) and 0.1% (v/v) trifluoroacetic acid/acetonitrile (Buffer B).

### Mass spectrometric analysis

Lyophilized HPLC fractions were dissolved in 0.1% (v/v) trifluoroacetic acid/water. 0.5 µl sample was spotted onto a matrix-assisted laser desorption ionization time-of-flight (MALDI-TOF) plate with 0.5 µl α-cyano-4-hydroxycinnamic acid matrix (10 mg/ml in 60% acetonitrile). Spots were analyzed by an UltraFlex I mass spectrometer (Bruker Daltonics) in a positive ion mode.

### Peptide sequencing

Partial amino acid sequence of antibacterial peptide was determined by Edman degradation using a pulsed liquid-phase Procise® Sequencer, Model 491 (Applied Biosystems, CA, USA).

### cDNA synthesis

Total RNA was extracted from the skin of salamanders using TRIzol (Life Technologies Ltd.) and then used for cDNA synthesis as described in our previous work [13]. The SMART™ PCR cDNA synthesis kit was purchased from Clontech (Palo Alto, CA). Two primers (3′SMART CDS PrimerII A, 5 ′-AAGCAGTGGTATCAACGCAGAGTACT (30) N-1N-3′ (N = A, C, G, or T; N-1 = A, G, or C), and SMART II An oligonu-cleotide, 5′-AAGCAGTGGTATCAACG CAGAGTACGCGGG-3′) were used to synthesize the first strand. 5′ PCR primer II A, 5′-AAGCAGTGGTATCAACGCAGAGT-3′ was used to synthesize the second strand using Advantage polymerase. All of these primers and polymerase are provided by the SMART™ PCR cDNA synthesis kit.

Rapid Amplification of cDNA ends (RACE) was used to clone transcripts encoding antibacterial peptide from the cDNA library [14]. Primers were designed according to the amino acid sequence determined by Edman degradation. The primers pairs 5′-RCANCCCCANACNGCRAA-3′ and 5′-AAGCAGTGGTATCAACG CAGAGTACGCGGG-3′ were used to determine mature peptide of CFBD-1. The signal peptide of CFBD-1 was determined by RACE using the primers pairs 5′-MGNGGNTAYTGYMGNGCN-3′ and 5′-AAGCAGTGGTATCAACGCAGAGT-3′ (N = A, C, G, or T; M = A or C; R = A or G; Y = C or T). The amplification conditions were set as follows: initial denaturation at 95°C for 2 min, followed by 34 cycles of denaturation at 92°C for 10 sec, annealing step at 52°C for 30 sec, extension step at 72°C for 40 s and a final elongation at 72°C for 10 min.

### Phylogenetic analysis

Sequences were aligned using ClustalW (Version 1.82). The defensin sequences were obtained from National Center for Biotechnology Information (NCBI) based on the blast results. A phylogenetic analysis was performed by using the software package MEGA 4.0. Bootstrap analysis (1000 replications) was used to evaluate the topology of the neighbor-joining tree.

### Antimicrobial testing

All microorganisms used for antimicrobial assays were obtained from Kunming Medical College. Microorganisms including Gram-positive bacterium *Staphylococcus aureus* (ATCC 25923), Gram-negative bacteria *Escherichia coli* (ATCC 25922), *Bacillus subtilis* (ATCC 6633), and fungus *Candida albicans* (ATCC 20032) were first grown in LB (Luria–Bertani) broth or yeast extract–peptone–dextrose broth as our previous methods [13]. Minimal inhibitory concentration (MIC) of tested sample against these microorganisms was determined as previous reports [15]. It is defined as the lowest concentration of tested sample to inhibit microorganism growth.

### Hemolytic assays

Hemolytic abilities were tested by human and rabbit red cells in Alsever's solution (in g/L: NaCl, 4.2; citric Acid · 3Na · 2H_2_O, 8.0; citric Acid · H_2_O, 0.55; D-glucose, 20.5) as reported [16]. Whole blood was mixed with Alsever's solution (V∶V 1∶1) and centrifuged at 1000 g for 5 min. The supernatant was discarded and the precipitated red cells were washed three times by Alsever's solution. The tested sample was serially diluted by Alsever's solution and incubated with washed red cells (10^8^ ml) at 37°C for 30 min. Red cells were centrifuged at 1000 g for 5 min before the absorbance of the supernatant was measured at 540 nm. 100% hemolysis was determined by adding 1% Triton X-100 to washed red cells.

## Results

### Peptide purification

**Figure 1:**
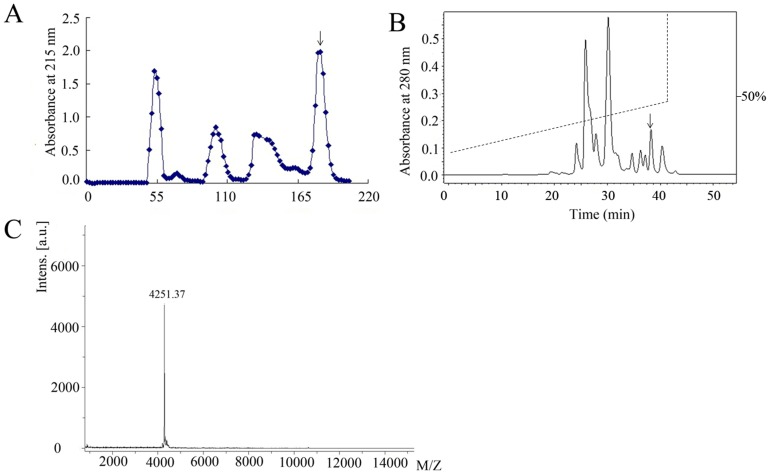
Purification of CFBD from salamander skin secretions of *C. fudingensis*. (A) Sephadex G-50 gel filtration of *C. fudingensis*. The elution was performed with 0.1 M phosphate buffer, pH 6.0, each collection of 3.0 ml. (B) Fraction marked by an arrow from the Sephadex G-50 gel filtration was further purified using RP-HPLC. Dashed line indicates linear gradients of acetonitrile. (C) Molecular mass of CFBD detected by mass spectrometry is 4251.37 Da.

The skin secretions of *C. fudingensis* were applied to Sephadex G-50 gel filtration. The elution was performed with 0.1 M PBS, collecting fractions of 3.0 ml/10 min by an automatic collector. As illustrated in Fig. 1A, the secretions were fractionated into six peaks. The last peak containing antimicrobial activity (marked by an arrow) was further purified using reverse-phase HPLC with a gradient of 10–50% buffer B over 40 min. The peptide (CFBD) with antimicrobial activity was found in the marked fraction (Fig. 1B).

### Sequence of CFBD-1

**Figure 2:**
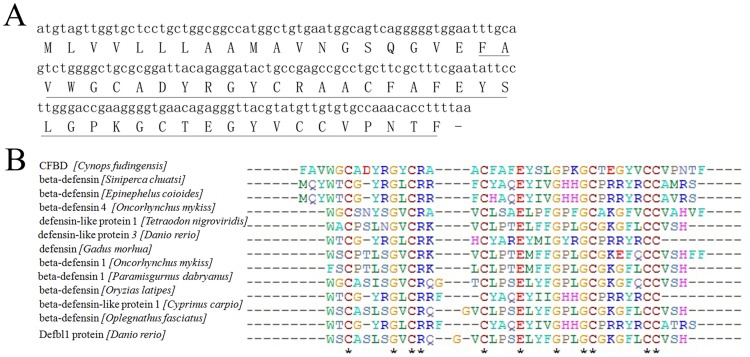
cDNA sequence and multiple sequence alignment of CFBD with other β-defensins. (A) The cDNA sequence of CFBD. The amino acid of the precursor reading from the cDNA is suggested below the nucleotide sequence. The sequence of mature peptide was underlined by solid line. (B) Alignment of CFBD with other β-defensins. Sequences of peptide were based on BLAST search results. The star (*) indicated the identical amino acid residue.

The amino acid sequence of CFBD-1 was determined by a combination method of automated Edman degradation and cDNA sequencing. Edman degradation gave the N-terminal sequence of FAVWGCADYRGYCRA. In order to obtain the full sequence of CFBD-1, rapid amplification of cDNA ends (RACE) was used to clone the transcripts from the cDNA library. As shown in Fig. 2A (GenBank accession: KF758841), the open frame encoded a 60 residues peptide precursor containing a signal peptide of 17 residues, a mature peptide of 41 residues and an intervening propeptide of 3 residues (Fig. 2A).The N-terminal sequence of mature peptide was completely consistent with the Edman degradation sequencing result of CFBD-1. The full amino sequence of the peptide is FAVWGCADYRGYCRAACFAFEYSLGP KGCTEGYVCCVPNTF. There are 41 residues including 6 cysteines, 3 acidic and 3 basic residues. Analysis using the ExPASy MW/pI tool (http://www.expasy.ch/tools/pi tool.html) showed that CFBD had a predicted isoelectric point (pI) of 6.14.

MALDI-TOF mass analysis indicated the molecular mass of CFBD-1 was 4521.37 Da (Fig. 1C). It is about 6 Da lower than the calculated mass (4527.18 Da), suggesting that three disulfide bridges were formed by six cysteines in the mature peptide. An online BLAST search showed that the mature peptide of CFBD-1 had significant sequence similarity to β-defensin antimicrobial peptides from other animals. As showed in Fig. 2B, CFBD-1 contains a conservative cysteine motif as found in other beta-defensins. Five additional residues (including 3 glycines, 1 arginine and 1 glutamate) are also conserved (Fig. 2B).

### Phylogenetic analysis

**Figure 3:**
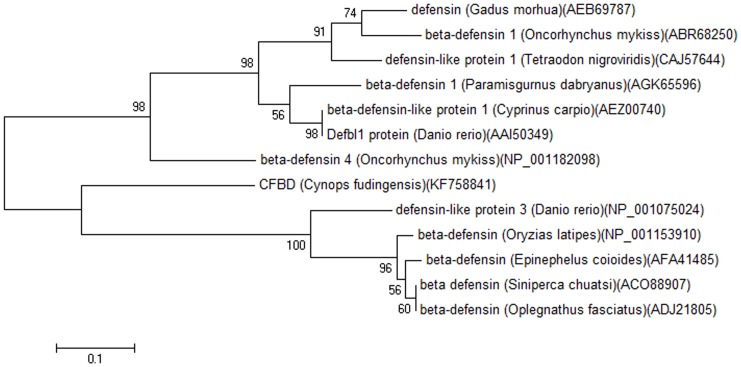
Phylogenetic analysis of CFBD with other β-defensins. Phylogenetic dendrogram obtained by neighbour-joining analysis based on the proportion difference (p-distance) of aligned amino acid of the full-length peptide sequences. All defensin sequences are from NCBI and the sequence ID is listed behind.

Evolution analysis was based on the multi-sequence alignments of mature peptides by MEGA software. The sequences of those peptides were clustered to construct a phylogenetic tree separately using ClustalW. As showed in Fig. 3, all the defensins are divided into two distinct clusters and CFBD-1 is most closely related to defensin-like protein 3 from *Danio rerio* (Fig. 3).

### Antimicrobial activities

Antimicrobial activity of CFBD-1 against Gram-positive, Gram-negative bacteria and fungi was tested. As listed in table 1, it exhibited weak antimicrobial activities against all the tested microorganisms. Of the tested strains, *S. aureus* (ATCC 25923) was the most sensitive to CFBD with a MIC of 65 µg/ml. The MIC of CFBD-1 against *B. subtilis* (ATCC 6633), *C. albicans* (ATCC 20032) and *E. coli* (ATCC25922) was 135, 200 and 160 µg/mL, respectively.

**Table 1:** Antimicrobial activity of β-defensin CFDB.

Microorganisms	MIC (µg/ml)
*Staphylococcus aureus* ATCC 25923	65
*Bacillus subtilis* ATCC 6633	135
*Candida albicans* ATCC 20032	200
*Escherichia coli* ATCC25922	160

Minimal peptide concentration required for total inhibition of cell growth in liquid medium; these concentrations represent mean values (±20%) of three replicates.

### Hemolytic activity

Human red blood cells and rabbit red blood cells were used to evaluate the hemolytic activity of CFBD-1. A little hemolytic activity was detected in our experiment. At the concentration of 400 µg/mL, CFDB-1 could induce about 2.5% and 3.2% of human red blood cells and rabbit red blood cells hemolysis, respectively.

## Discussion

Antimicrobial peptides on salamander skin may act as a part of the innate immune system of salamanders. The innate immune system makes them surviving well in a variety of conditions laden with pathogenic microbes. Little work has been done about salamander antimicrobial agents. In this study, the first defenisn antimicrobial peptide (CFBD) was identified from the salamander skin of *C. fudingensi*. CFDB exhibited moderate antimicrobial activity against the Gram-positive *S. aureus*.

β-defensin antimicrobial peptides have been found in most of vertebrates. Many amphibian antimicrobial peptide families containing disulfide-bridged segment were identified such as nigrocin, brevinin, esculentin, odorranain-C, -H, -P [17], [18], but defensins family were seldom detected. The first and only amphibian-encoded β-defensin was identified from the Chinese brown frog, *Rana chensinensis* [19]. Up to now, no β-defensin was isolated from urodela amphibian. The mature peptide of CFBD also contained three disulfide-bridges and showed significant sequence similarity with β-defensin antimicrobial peptides, especially six conserved cysteines. It suggested that CFBD should be classified as a new member of β-defensin antimicrobial peptide family.

The evolution analysis of revealed that CFBD is the most close to β-defensin from zebrafish *D. rerio* (Fig. 3). Fish defensins were discovered though many species such as zebrafish (*D. rerio*), green-spotted pufferfish (*Tetraodon nigroviridis*), the tiger pufferfish (*Takifugu rubripes*) and rainbow trout (*Oncorhynchus mykiss*) [20], [21]. Biological functions of fish beta-defensin have been investigated only in a few fish species [22], [23] and they could inhibit Gram-positive and Gram-negative bacteria implying that antimicrobial peptides such as defensins comprise the first line of defense to clear pathogens rapidly in the evolutionary process. CFBD showed moderate activity to Gram-positive *S. aureus*, while weak antimicrobial abilities against other microorganism strains. It was similar to another salamander antibacterial peptide F15, which exhibited activity to *S. aureus* but not other bacteria [17].

The identification of β-defensin-like peptide CFBD in the salamander demonstrated that there is the presence of β-defensin antimicrobial peptide in urodela amphibians. More work is necessary to identify antimicrobial agents from urodela amphibians and to understand the immune system of salamanders.
